# The Efficacy of Scalp Nerve Block in Postoperative Pain Management after Microvascular Decompression: A Randomized Clinical Trial

**DOI:** 10.3390/jcm12134242

**Published:** 2023-06-24

**Authors:** Eun Kyung Lee, Seungwon Lee, Ji-Hye Kwon, Seung Hoon Lee, Soo Jung Park, Yunghun Kim, RyungA Kang, Ji Seon Jeong, Jeong Jin Lee

**Affiliations:** 1 Department of Anesthesiology and Pain Medicine, Chung-Ang University College of Medicine, Seoul 06973, Republic of Korea; 2 Department of Anesthesiology and Pain Medicine, Samsung Medical Center, Sungkyunkwan University School of Medicine, Seoul 06351, Republic of Korea; 3 Department of Neurosurgery, Samsung Medical Center, Sungkyunkwan University School of Medicine, Seoul 06351, Republic of Korea; 4 Department of Anesthesiology and Pain Medicine, Ajou University School of Medicine, Suwon 16499, Republic of Korea

**Keywords:** scalp nerve block, anesthesia, enhanced recovery after surgery, microvascular decompression, hemifacial spasm

## Abstract

The scalp nerve block, created by injecting local anesthetics around the scalp nerves, is reported to effectively reduce pain after surgery. In this study, we evaluated the efficacy of scalp nerve block in patients with hemifacial spasm (HFS) undergoing microvascular decompression (MVD). Seventy-four patients who underwent MVD for HFS were enrolled. The block group received scalp nerve block with 0.5% ropivacaine before surgery. The primary outcome was cumulative dose of rescue analgesics 24 h postoperatively. The secondary outcomes were included pain scores, postoperative antiemetic consumption, and Quality of Recovery-15 scale. The cumulative dose of rescue analgesics at 24 h postoperatively was not significantly different between the two groups (4.80 ± 3.64 mg vs. 5.92 ± 3.95 mg, *p* = 0.633). However, the pain score was significantly reduced in the block group at 6, 12, and 24 h postoperatively. Postoperative antiemetic consumption was lower in the block group than the control group at 12 h. There were no significant differences between the two groups for other secondary outcomes. In MVD for HFS, a preoperative scalp nerve block might reduce postoperative pain in the early postoperative period, but a larger study using a multimodal approach is needed to confirm the efficacy of a scalp block.

## 1. Introduction

Microvascular decompression (MVD) is a surgical procedure to treat hemifacial spasm (HFS) and trigeminal neuralgia, which are caused by vascular compression of cranial nerve VII [1,2]. As a surgical procedure, an incision is made in the postauricular area [3], which can damage micronerves in the scalp. Because many nerves are concentrated in that area, the pain immediately after surgery can be distressing, and one-quarter of patients suffer from subsequent chronic pain [4]. Previous studies have focused on MVD as a treatment for trigeminal neuralgia [5,6] and found that satisfactory postoperative pain management can improve the quality of recovery and prevent transition to chronic pain after surgery [4,7,8,9]. However, few studies have considered postoperative pain management in patients undergoing MVD for HFS, which is especially important because the symptom of HFS is myoclonic muscle spasm without pain, and the goal of MVD is to improve patient quality of life [2,10,11,12]. Considering that HFS patients are typically in a pain-free state prior to surgery, the occurrence of postoperative pain can have an unforeseen impact on their recovery. Perioperative pain management is particularly important in this population.

Enhanced recovery after surgery (ERAS) and its multimodal interventions have created a paradigm shift in postsurgical care, including in neurosurgery. Scalp nerve block has been introduced and implemented in various neurosurgeries to stabilize surgical procedures, manage postoperative pain, and reduce opioid consumption [13,14,15]. The ERAS protocol for elective craniotomy recommends the use of a scalp block in addition to NSAID [16,17,18,19,20], but evidence on this topic is lacking. Therefore, in this study, we evaluated the efficacy of a regional scalp nerve block in HFS patients undergoing MVD via the lateral suboccipital approach, a region associated with severe postoperative pain. 

We hypothesized that the scalp block with ropivacaine would reduce the postoperative opioid dosage during the first 24 h after surgery.

## 2. Materials and Methods

This study was a two-group, parallel, randomized, and controlled clinical trial conducted at a tertiary medical center. The Samsung Medical Center Institutional Review Board (IRB) approved this study (approval number: SMC 2020-10-142), and the trial was registered at the Clinical Research Information Service (identifier: KCT0005846; principal investigator: Jeong Jin Lee; registration date: 28 January 2021). Written informed consent was obtained from each patient one day before their participation in the study. All methods were conducted in accordance with the principles of the Declaration of Helsinki and Good Clinical Practice Guidelines.

### 2.1. Patient Enrolment

Adult patients (20–70 years old) undergoing elective MVD to treat HFS were enrolled from 29 January 2021 to 7 January 2022. Patients with hypersensitivity or adverse reactions to local anesthetics or psychiatric disease before surgery, the use of antidepressant medication, a creatinine level more than 2.0 mg/dL, platelet count < 50,000/µL, activated partial thromboplastin time > 40 s, and prothrombin time (international normalized ratio) > 1.5 were excluded from the study. Other exclusion criteria were chronic pain or a history of drug abuse. Chronic pain was defined as pain lasting longer than three months or requiring analgesics for an extended period. To ensure no history of drug abuse, the Drug Utilization Review provided by Health Insurance Review and Assessment service of Korea was searched for each patient. Additionally, all patients underwent interviews to further confirm their history of drug use or abuse.

### 2.2. Randomization and Blinding

Seventy-four patients were randomly allocated to the scalp nerve block group (block group) or control group using computer-generated block randomization (www.randomizer.com, accessed on 1 October 2020) with an allocation ratio of 1:1 in blocks of 4. IRB disagreed with the sham procedure, concerning ethical issues. Group allocation was not blinded to the block provider or patients, but the surgeon, anesthetic providers, and postoperative outcome assessor were blinded.

### 2.3. Intervention

**Figure 1:**
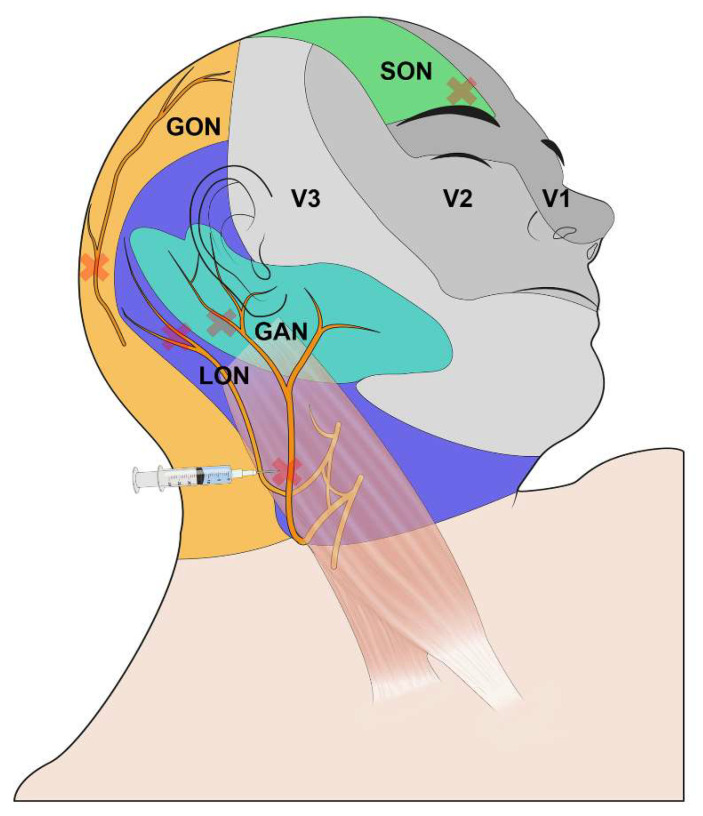
Scalp nerve block. The colored area represents the dermatome of the corresponding nerve. The x marks indicate sites of local anesthetic injection. Contralateral GON, which could not be shown in the figure, was also injected. Abbreviations: GAN, great auricular nerve; GON, greater occipital nerve; LON, lesser occipital nerve; SON, supraorbital nerve; V1: Ophthalmic nerve of the fifth cranial nerve; V2: Maxillary nerve of the fifth cranial nerve; V3: Mandibular nerve of the fifth cranial nerve.

Patients underwent standard monitoring for oxygen saturation, electrocardiography, noninvasive blood pressure, neuromuscular changes, and bispectral index (BIS). Before induction into the block group, the patients were premedicated with midazolam, and the following series of blocks was performed by an independent anesthesiologist. The skin was sterilized with 2% chlorhexidine after protecting the patient’s eye with gauze. An ultrasound-guided or blinded scalp nerve block using a 26-gauge needle was performed [21] (Figure 1). For the local anesthetic, 0.5% ropivacaine was prepared. For patients without cardiovascular disease such as history of percutaneous coronary intervention or angina, epinephrine 1:200,000 was added to the local anesthetic. The first step was to block the surgical site of the supraorbital nerve with 2 mL of anesthetic while the patient was in the supine position to alleviate pain at the head fixation pinning site. Next, the patient was asked to turn their head in the contralateral direction of the surgical site, and the midpoint of the posterior border of the clavicular head of the sternocleidomastoid muscle was marked. The great auricular nerve was explored using ultrasound and was blocked using the long-axis in-plane technique. Finally, as the patient leaned their body more toward the contralateral direction, the lesser and bilateral greater occipital nerves were identified and blocked under ultrasound guidance. The total amount of ropivacaine injected ranged from 21 to 25 mL. When the intervention was complete, the sensory blockade was assessed in the forehead, upper neck, and occipital regions. Block success was defined as loss of sensation to cold stimuli at all sites. Induction of general anesthesia began after the block check was complete. Any complication of the block (bleeding, hematoma, or nerve injury) was assessed and recorded immediately after the procedure.

### 2.4. Anesthetic Management

Anesthesia was induced with propofol (effect site target-controlled infusion (TCI) of 5 µg/mL) and remifentanil (effect site TCI of 3 ng/mL). For the effect-site TCI, a commercial TCI pump (Orchestra Base Primea, Fresinus Vial, France) was used. After loss of consciousness, 0.6–0.8 mg/kg of rocuronium was administered intravenously, and the patient was manually ventilated with 100% oxygen. After confirming neuromuscular blockade with a train-of-four (TOF) count of 0, tracheal intubation was performed with a 7.0 mm (internal diameter) endotracheal tube (ETT) for women and 8.0 mm ETT for men, and cuff pressure was adjusted to between 20 and 25 mm H_2_O with a hand pressure gauge. After intubation, supplementary monitors were applied to measure end-tidal carbon dioxide (EtCO_2_), esophageal temperature, and invasive arterial blood pressure through a radial artery catheter. Anesthesia was continued with an effect-site TCI of propofol and remifentanil to control BIS to a target level of hypnosis of 40 to 60 and blood pressure and heart rate to within 20% of their baseline values. Hypotension (baseline mean arterial pressure < 20%) was treated with 5 mg of ephedrine, and bradycardia (baseline heart rate < 20%) was treated with 0.5 mg of atropine. Hypertension (baseline mean arterial pressure > 20%) was treated with 300 μg of nicardipine. Mechanical ventilation was maintained with a tidal volume of 8 mL/kg, and ventilator frequency was controlled to maintain an EtCO_2_ between 35 and 40 mmHg. Patient body temperature was controlled at a target value of 36.5 °C. After completion of the procedure, 0.5 mg/kg of pethidine was administered, and self-respiration was recovered with an injection of 2–4 mg/kg of sugammadex as determined by the TOF value to reverse the neuromuscular block. The patient was transferred to the intensive care unit (ICU) in an intubated state with spontaneous respiration following standard protocol in our center. Every procedure except the scalp nerve block was the same between the two groups.

### 2.5. Postoperative Management

Patients were kept in the ICU overnight after surgery, and postoperative assessments were performed at 6, 12, and 24 h. Postoperative analgesia was standardized at 1 g of intravenous acetaminophen every 8 h. In addition, 30 mg of intravenous ketorolac was administered when patients reported pain of 4 or 5 on the numeric rating scale (i.e., NRS; 0–10; 0 = no pain and 10 = worst possible pain), and 25 mg of intravenous pethidine was given for pain reported as a 6 or higher. For shivering, 25 mg of intravenous pethidine was administered, and “shivering” was noted on the electrical medical record. Postoperative nausea and vomiting (PONV) is categorized into four levels: none, mild, moderate, and severe. A mild level is characterized as tolerable nausea. A moderate level is defined as having vomited once since the last assessment, and the severe level is indicated by multiple episodes of vomiting. As a regular antiemetic, 0.3 mg of ramosetron was provided every 12 h. For moderate to severe PONV, 10 mg of metoclopramide was provided as a rescue antiemetic.

### 2.6. Outcome Variables

The primary outcome was the total opioid consumption within 24 h after surgery, converted into morphine milligram equivalents (MME). The calculation involved multiplying the milligrams (mg) of intravenous pethidine by 10 and dividing the result by 75 [22]. Because the same dose of pethidine was used for postoperative pain and shivering, the nonshivering MME was also calculated. Secondary outcomes were MME at 6 and 12 h after surgery, NRS of pain, severity of PONV, cumulative dose of nonopioid analgesics, cumulative dose of antiemetics, any side effects of the analgesics at each assessment, the total dose of anesthesia drugs used (propofol, remifentanil, and pethidine), the use of ephedrine or nicardipine, duration of anesthesia induction, duration of anesthesia, duration of surgery, time to first rescue opioid, and maximum NRS of pain during the first 24 h. The area under the curve (AUC) of the pain NRS was calculated by assuming equal intervals between assessments, even though the intervals were different. Complications from the block (bleeding, hematoma, and nerve injury) and vital signs were assessed immediately after the procedure and at 6, 12, and 24 h after surgery. 

In addition, the score for the Korean version of the Quality of Recovery-15 (QoR-15K) [23] was obtained before surgery and 24 h after surgery. The QoR-15K is a self-reported questionnaire for Korean speakers that contains 15 items to assess patient quality of recovery and emotional status following surgery. It is composed of 5 categories: physical comfort, emotional status, psychological support, physical independence, and pain. The patient scores each question from 0 to 10, and the sum of all items ranges from 0 to 150, with a higher score indicating better recovery.

### 2.7. Statistical Analysis

A reduction of 30% in total opioid consumption during the first 24 h after surgery was considered a clinically significant difference according to previous neurosurgical studies [24,25]. The sample size was calculated based on our preliminary data. With an MME for 24 h of 6.4 ± 2.7 mg (mean ± standard deviation (SD)), alpha error of 0.05, and power of 0.8, 33 participants were required for each group. Considering a dropout rate of 10%, we planned to enroll a total of 74 patients. All continuous data were tested for normality using the Shapiro–Wilk test and visual inspection of a histogram. Data are presented as mean ± SD, median (interquartile range [IQR]), or number (percentage). Differences between groups were analyzed using the chi-square test for categorical variables and Student’s *t*-test or Wilcoxon’s rank sum test for continuous variables. The Bonferroni correction was applied for multiple comparisons. The AUC for the pain NRS was also calculated. A *p*-value less than 0.05 was considered statistically significant. Statistical analyses were carried out using SPSS (version 27.0; SPSS Inc., Chicago, IL, USA) and GraphPad Prism (Version 5.0, GraphPad Software Inc., San Diego, CA, USA).

## 3. Results

### 3.1. Study Participants

**Figure 2:**
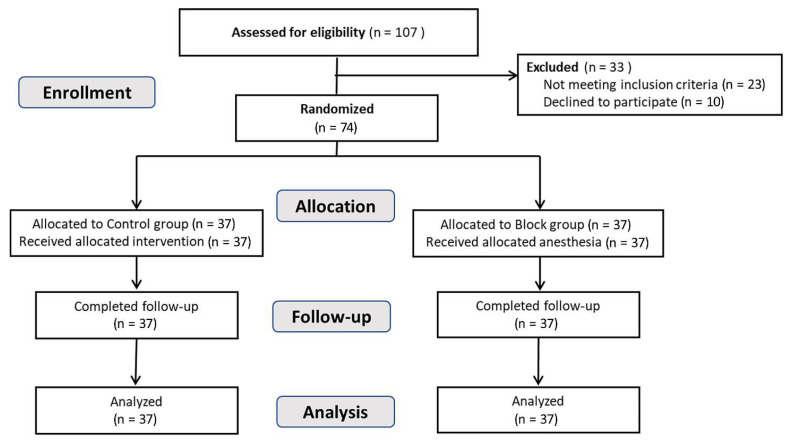
Flow diagram indicating the consolidated standards of reporting trials for patients included in the study.

We assessed 107 patients for study eligibility, and 33 of those patients met the exclusion criteria or declined to participate. All enrolled patients (*n* = 74) were randomly assigned to the control group or the scalp nerve block group (37 each). No patient in the scalp nerve block group was identified to have pre-existing cardiovascular diseases; hence, ropivacaine was administered along with epinephrine to all patients in the block group. All 74 patients completed the follow-up procedures and were included in the final analysis (Figure 2). All scalp nerve blocks conducted in the block group met the conditions for success at 5 min after block completion, and no patient experienced side effects after the nerve block. The baseline demographic and clinical characteristics of the two groups are shown in Table 1. No patients had bilateral HFS, and the number of left-sided HFS patients was 22 (59.5%) and 19 (51.4%) in the block group and control group, respectively.

Table 2 shows the intraoperative parameters in the two groups. In the block group, the duration of induction was significantly longer than in the control group. Other intraoperative variables (duration of anesthesia; duration of surgery; total dose of propofol, remifentanil, and pethidine; and use of ephedrine and nicardipine) did not differ statistically. No nerve-block-related or anesthesia-related complications occurred in any of the participants during hospital stay. Excluding the one patient in the block group who required prolonged intubation, the median and interquartile range of extubation time from the end of surgery was 35 (26, 42) minutes in the control group and 29 (23, 40) minutes in the block group (*p*-value = 0.185). One patient who underwent prolonged intubation for 1160 min experienced intraoperative bleeding, necessitating sedation on the day of surgery. The following morning, after confirming stable vital signs and neurological status, extubation was performed.

### 3.2. Primary Outcome and Opioid Consumptions

**Figure 3:**
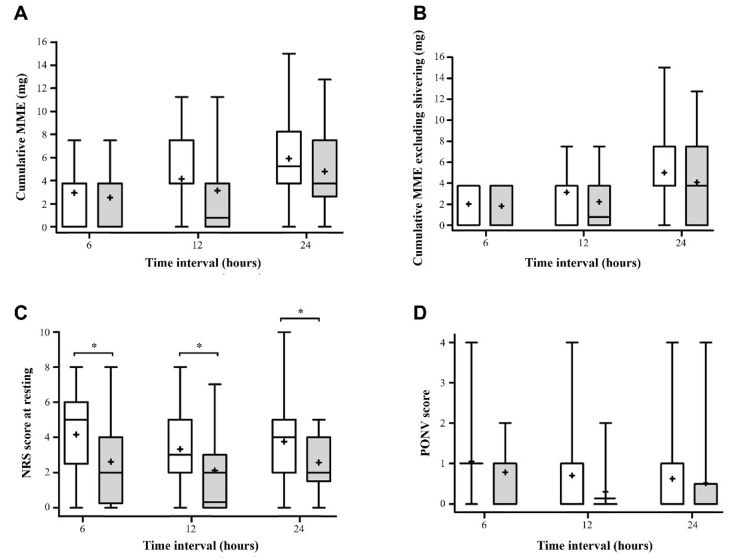
Comparison of box and whiskers plots between the control (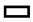) and block (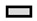) groups for (**A**) cumulative MME, (**B**) cumulative MME excluding shivering, (**C**) the NRS of pain at rest, and (**D**) PONV at 6, 12, and 24 h postoperatively. The solid lines in the box indicate medians, the plus signs (+) in the box indicate mean, the boxes indicate interquartile ranges, and the whiskers indicate minimum to maximum. * significant difference between the two groups by Student’s *t*-test or Wilcoxon’s rank sum test (*p* < 0.05) in accordance with the Bonferroni correction. MME, morphine milligram equivalents; NRS, numeric rating scale; PONV, postoperative nausea and vomiting.

The mean MME were 2.94 ± 2.00 vs. 2.53 ± 1.99 (*p*-value > 0.99) at 6 h; 4.16 ± 2.76 vs. 3.14 ± 2.42 (*p*-value = 0.294) at 12 h; and 5.92 ± 3.95 vs. 4.80 ± 3.64 (*p*-value = 0.633) at 24 h in the block group and control group, respectively. The difference was not statistically significant (Figure 3A). Opioid consumption excluding that for shivering at 6, 12, and 24 h failed to achieve statistical significance (2.03 ± 1.89 vs. 1.82 ± 1.90, *p*-value = 0.647 at 6 h; 3.14 ± 2.87 vs. 2.23 ± 2.25, *p*-value = 0.132 at 12 h; 5.01 ± 3.79 vs. 4.09 ± 4.09, *p*-value = 0.323 at 24 h) but tended to be lower in the block group (Figure 3B). Though not originally planned in the study protocol, a chi-square test was conducted to assess the administration of pethidine based on its distribution (Table 3). There were no significant differences between the two groups. Similarly, when performing the Mann–Whitney test for the number of pethidine administrations, there were no significant differences observed (Table 3). The time to the first rescue opioid was 80 (31, 208) minutes and 55 (32, 120) minutes (median (IQR)) for the block group and control group, respectively (*p*-value = 0.492). The time to first rescue opioid excluding that for shivering was 124 (45, 227) minutes and 64 (44, 144) minutes for the block group and control group, respectively (*p*-value = 0.489). The dose of ketorolac was lower in the block group than the control group at every assessment, but statistical significance was not observed (Table 3). The dose of regular acetaminophen did not differ between the groups (Table 3). 

### 3.3. Secondary Outcomes

The pain scores at 6, 12, and 24 h postoperatively were significantly lower in the block group than the control group (Table 3, Figure 3C). The maximum NRS for the 24 h period was statistically lower in the block group than the control group (Table 3). The overall pain score using the AUC was also lower in the block group (Table 3).

The PONV grade did not differ significantly between the groups (Figure 3D). However, the use of rescue antiemetics was significantly lower in the block group than the control group at 12 h (Table 3).

The QoR-15K at 24 h and length of hospital stay did not differ significantly between the groups (Table 3).

## 4. Discussion

In this single-center, randomized clinical study, we observed changes in MME, pain score, and complications during the first 24 h after surgery in the scalp nerve block group versus the control group. Although postoperative opioid consumption did not differ statistically between the groups, the postoperative pain scores for all the observation periods were significantly lower in the block group. The mean difference between the control group and block group decreased over time, which implies that the scalp block was effective, and it is necessary to find a way to extend the block duration. Additionally, we found that the maximum NRS during the first 24 h after surgery was lower in the block group than the control group, which has clinical relevance. Furthermore, the AUC of NRS pain for the whole 24-h period also showed clear differences between the groups, which highlights the potential benefits of scalp nerve block as a pain management strategy in the postoperative period. 

Previous studies have also observed that a scalp block decreases pain scores for up to 12 h after surgery, and although an overall reduction in the opioid requirements has been observed, the studies have shown some heterogeneity [15,26]. We acknowledge that the question may rise regarding the justification for our hypothesis targeting only the first 24 h after surgery and not a longer duration. The rationale behind our decision was primarily based on the immediate postoperative period being critical for effective pain management and patient comfort. Previous studies have indicated that the highest intensity of postoperative pain is typically experienced within the first 24 h after surgery, and effective pain control during this period can significantly impact patient recovery, satisfaction, and overall outcomes [15,24]. By assessing the efficacy of scalp nerve block within this early postoperative period, we aimed to evaluate its immediate analgesic effects and provide valuable insights into the potential benefits of this intervention in enhancing early pain relief following microvascular decompression. ERAS protocols for craniotomy have recently been developed [16,17,27], and a scalp block is one of the main anesthetic elements. As Wang et al. emphasize [20], effective pain control using a scalp nerve block after MVD is important not only for analgesia, but also to improve the quality of recovery. Although the induction time of the block group was 5.7 min longer than in the control group, it did not lengthen the overall surgery time or anesthetic time. The preoperative nerve block did not reduce the remifentanil dose during surgery, unlike the result in a previous study [28]. 

In this study, the great auricular nerve is included for scalp nerve block. Prior research on scalp nerve blocks have primarily focused on the application of occipital nerve blocks for posterior craniotomy and trigeminal nerve blocks for anterior craniotomy [14,15]. However, MVD involves a lateral approach from behind the ipsilateral ear, and it requires an additional nerve block to address the anatomical region involved. The great auricular nerve, originated from the cervical plexus, provides sensory innervation to the postaural area. By additionally targeting the great auricular nerve, effective pain reduction and improved patients’ quality of recovery were expected perioperatively.

The mean MME at 24 h decreased by 1.12 mg in the block group compared with the control group; however, that was not a statistically significant improvement in opioid consumption. The primary outcomes in both groups were lower than the value obtained from the preliminary study, which was used in sample size calculation. Following the prior study, acetaminophen was administered regularly in ICU, potentially resulting in a decrease in the overall opioid consumption. Both MME were lower than the MME reported in previous studies [24,25]. However, those studies involved craniotomies that were not specific to MVD, and recent advances in perioperative management might have affected the results. On the other hand, the MME reported here are comparable to other studies [26,29]. In our results, the MME at 6 h showed the smallest difference between groups (0.41 at 6 h, 1.02 at 12 h, and 1.12 at 24 h). The difference at 6 h might be attenuated because 0.5 mg/kg of pethidine was administered after completion of the procedure in all patients. Before this study, 25 mg of pethidine was generally injected when a post-MVD patient arrived in the ICU because some patients were agitated or shivering immediately after surgery. A further study using analgesia nociception index would provide a better understanding of the relationship between the block and intraoperative pain. Considering the mean anesthetic time and median remifentanil dose, the effect of a scalp block on MVD is promising. For analgesics, we used weight-based opioids during surgery and a fixed dose of opioid in the ICU. It would be ideal to administer analgesics individually, but administering a uniform dose of analgesic in the ICU or ward is more practical and widely used. Given these limitations, a meta-analysis is expected to thoroughly access the impact of scalp nerve block on MVD.

This study has limitations. First, due to shivering after surgery, pethidine was administered to patients in both groups. The amount injected due to shivering was excluded from the calculation. Even though the amount of pethidine was minimal, its use for nonpain control could be considered a limitation of this study. However, the groups did not differ significantly in the use of pethidine for postoperative shivering. Second, patients could not be blinded to their block condition because we had to confirm that the block was performed adequately. The block was thus performed before general anesthesia induction for the wound area and pin placement during surgical manipulation. Because the average length of anesthesia time was about 3 h in both groups, a further study to extend the block duration is encouraged. Last, the sample size was calculated to detect differences in MME; thus, differences in secondary outcomes were not detected.

It is important to approach the interpretation of the use of antiemetics at 12 h with caution. While there was a significant reduction in the use of antiemetics at 12 h in the block group compared to the control group, no significant difference was observed in the severity of PONV at the same time point. Additional research is necessary to explore the effect of scalp nerve block on PONV.

## 5. Conclusions

In MVD for HFS, a preoperative scalp nerve block could reduce postoperative pain in the early postoperative period, although it did not significantly decrease postoperative opioid consumption. A further study with a larger population is needed to confirm the efficacy of a scalp nerve block from the perspective of ERAS.

## Figures and Tables

**Table 1:** Patient baseline characteristics.

Parameter	Control Group (*n* = 37)	Block Group (*n* = 37)
Sex (female)	26 (70.3%)	21 (56.8%)
Age (year)	51.0 (46.0, 58.0)	53.0 (44.0, 60.0)
Height (cm)	158.0 (153.6, 165.6)	164.5 (156.0, 172.0)
Weight (kg)	65.1 ± 11.2	67.2 ± 12.8
BMI (kg/m^2^)	25.5 ± 2.9	25.0 ± 3.2
ASA-PS		
I	12 (32.4%)	14 (37.8%)
II	25 (67.6%)	23 (62.2%)
Current smoker	5 (13.5%)	1 (2.7%)
Hypertension	7 (18.9%)	10 (27.0%)
Diabetes	3 (8.1%)	3 (8.1%)
Site of operation (left)	19 (51.4%)	22 (59.5%)
QoR-15K, preoperative	139.2 ± 12.8	137.2 ± 19.4

Values are mean ± standard deviation, median (interquartile range), or number (percentage). ASA-PS, American Society of Anesthesiologists physical status; BMI, body mass index; QoR-15K, Korean version of Quality of Recovery-15.

**Table 2:** Intraoperative parameters between the control group and scalp nerve block group.

Parameter	Control Group (*n* = 37)	Block Group (*n* = 37)	*p*-Value
Duration of induction (min)	14.1 ± 3.88	19.8 ± 6.77	<0.001 *
Duration of operation (min)	102 ± 19.2	106 ± 24.1	0.388
Pethidine (mg)	30.0 (25.0, 35.0)	30.0 (25.0, 35.0)	0.262
Propofol (mg)	1240.0 (1100.0, 1480.0)	1400.0 (1140.0, 1640.0)	0.36
Remifentanil (mg)	0.9 (0.8, 1.0)	0.8 (0.6, 1.1)	0.295
Use of ephedrine (n)	7 (18.9%)	14 (37.8%)	0.122
Use of nicardipine (n)	4 (10.8%)	3 (8.1%)	>0.99

Values are mean ± standard deviation, median (interquartile range), or number (percentage). * Significant difference between the two groups by Student’s *t*-test or Wilcoxon’s rank sum test (*p*-value < 0.05).

**Table 3:** Postoperative clinical outcomes between the control group and scalp nerve block group.

Outcome	Control Group (*n* = 37)	Block Group (*n* = 37)	*p*-Value
Pain score (NRS, 0–10)			
6 h postoperative ^§^	5 (3, 6)	2 (1, 4)	0.005 *
12 h postoperative ^§^	3 (2, 5)	2 (0, 2)	0.007 *
24 h postoperative	4 (2, 5)	2 (2, 4)	0.015 *
Max NRS for 24 h	7 (5, 7)	4 (2, 6)	<0.001 *
AUC over 24 h	7 (5.5, 9)	4 (2, 6)	<0.001 *
Patients requiring pethidine (n %)			
6 h	27 (73.0)	24 (64.9)	0.451 ^†^
12 h	30 (81.1)	27 (73.0)	0.407 ^†^
24 h	31 (83.8)	28 (75.7)	0.386 ^†^
Frequency of pethidine administration			
6 h	1 (0, 1)	1 (0, 1)	0.393 ^‡^
12 h	1 (1, 2)	1 (0, 1)	0.081 ^‡^
24 h	1 (1, 2)	1 (0.5, 2)	0.215 ^‡^
Patients requiring pethidine, nonshivering (n %)			
6 h	21 (56.8)	18 (38.6)	0.485
12 h	28 (75.7)	23 (62.2)	0.209
24 h	30 (81.1)	24 (64.9)	0.116
Postoperative ketorolac (mg)			
6 h ^§^	6.49 ± 12.52	5.68 ± 13.85	0.792
12 h ^§^	12.97 ± 19.42	7.30 ± 14.84	0.486
24 h	25.14 ± 28.73	13.78 ± 21.90	0.180
Postoperative acetaminophen (g)			
6 h	0.97 ± 0.37	1.03 ± 0.16	0.422
12 h	1.89 ± 0.52	1.97 ± 0.16	0.367
24 h	2.89 ± 0.81	3.11 ± 0.39	0.150
Postoperative nausea scores (0–4)			
6 h ^§^	8/21/7/0/1	11/22/3/0/0	0.337 ^†^
12 h ^§^	19/12/5/0/1	27/6/3/0/0	0.130 ^†^
24 h	23/8/4/1/1	28/4/1/3/1	0.308
Postoperative metoclopramide (mg)			
6 h ^§^	1.35 ± 3.47	0.54 ± 2.29	0.720
12 h ^§^	3.51 ± 5.88	0.81 ± 2.77	0.042 *
24 h	4.86 ± 7.68	2.43 ± 5.48	0.366
QoR-15K, postoperative	97.3 ± 32.9	107.0 ± 24.0	0.152
Hospital stay (days)	7.4 ± 1.0	7.2 ± 0.9	0.548

Values are mean ± standard deviation, median (interquartile range), or number (percentage). * Significant difference between the two groups by Student’s *t*-test or Wilcoxon’s rank sum test in accordance with the Bonferroni correction (*p*-value < 0.05). ^†^ chi-square tests were performed. ^‡^ Mann–Whitney U tests were performed. ^§^ In one patient in the block group, NRS and nausea scores at 6 and 12 h were not measured due to delayed extubation. NRS, numeric rating scale; QoR-15K, Korean version of Quality of Recovery-15.

## Data Availability

The data presented in this study are available on request from the corresponding author.
